# Bibliometric analysis of global Lassa fever research (1970–2017): a 47 – year study

**DOI:** 10.1186/s12879-018-3526-6

**Published:** 2018-12-10

**Authors:** Henshaw Uchechi Okoroiwu, Francisco López-Muñoz, F. Javier Povedano-Montero

**Affiliations:** 1 Haematology Unit, Department of Medical Laboratory Science, University of Calabar, Calabar, Nigeria; 2 Faculty of Health Sciences, University Camilo José Cela, Madrid, Spain; 3 Neuropsychopharmacology Unit, Hospital 12 de Octubre Research Institute (i+12), Madrid, Spain; 4 Portucalense Institute of Neuropsychology and Cognitive and Behavioural Neurosciences (INPP), Portucalense University, Porto, Portugal; 5 Thematic Network for Cooperative Health Research (RETICS), Addictive Disorders Network, Health Institute Carlos III, MICINN and FEDER, Madrid, Spain; 6 Faculty of Biomedical Sciences and Health, European University of Madrid, Madrid, Spain

**Keywords:** Lassa, Lassa fever, Lassa research, Bibliometric analysis

## Abstract

**Background:**

Lassa fever has been a public health concern in the West African sub-region where it is endemic and a latent threat to the world at large. We investigated the trend in Lassa fever research using bibliometric approach.

**Methods:**

We used the SCOPUS database employing “Lassa fever” as search descriptor. The most common bibliometric indicators were applied for the selected publications.

**Results:**

The number of scientific research articles retrieved for Lassa fever research from 1970 to 2017 was 1101. The growth of publications was more linear (*r* = 0.67) than exponential (*r* = 0.53). The duplication time of the scientific articles was 9.19 years. Small number of authors were responsible for bulk of the article production (transience index of 78.89%). The collaboration index was 4.59 per paper. The Bradford core consisted of 19 journals in which *Journal of Virology* was at the top (4.6%). Majority of the output were from USA government agencies. United States was the most productive country. Joseph B. McCormick was the most productive author, while *New England Journal of Medicine* published the two most cited articles.

**Conclusion:**

The growth of scientific Literature on Lassa fever was of linear pattern with high transient authors indicating low productivity and non-specialized authors from other related areas publishing sporadically. This study provides a helpful reference for medical virologists, epidemiologist, policy decision makers, academics and Lassa fever researchers.

## Background

Lassa fever is a viral hemorrhagic fever that was first described in the town Lassa in the North-East of Nigeria [1, 2]. Lassa virus (LASV), the causative agent of Lassa fever, is a negative strand RNA virus belonging to the old world complex family *Arenaviridae* characterized by the appearance of “sandy” ribosomes encapsulated in the virion as seen in electron microscope [3–5]. The reservoir/natural host of the virus is the multimammate rat *Mastomys natalensis* which live close to human settlement [4]. The virus may be transmitted from human to human giving rise to nosocomial or community-based outbreaks [5]. *Mastomys natalensis* shed the virus in urine [6] and contamination of human food is a more likely mode of transmission. Clinical manifestations of Lassa fever ranges from asymptomatic infection to hemorrhagic fever [7]. Approximately one-third of Lassa fever survivors develop bilateral or unilateral sudden-onset sensorineural hearing loss (SNHL) from which some patients fully recover [3, 8]. Lassa fever is endemic in West Africa causing an estimated 500,000 cases and 5000 deaths per year [3]. The countries implicated in the epidemic are Sierra Leone: Panguma and Kenema; Liberia: Zorzor, Phebe and Gianta; Nigeria: Jos, Onitsha, Zonkwa, Vom, Imo, Laffia, Irrua and Abakiliki [4, 9]. However, more recent oversea cross border infection has been reported in 2016 in Germany [10]. Lassa virus is a class 1A infective agent thus requiring a high-level containment, biosafety level 4 facility to diagnose or research [11].

Bibliometric studies are important tools for evaluating the social and scientific relevance of a given discipline within a specified time frame. The term “bibliometrics” was introduced in 1969 by Allan Pritchard, to define the use of mathematical and statistical procedures to the process of propagation of written communication in the field of scientific disciplines, via quantitative study of the varying aspect of this type of communication [12]. Despite the methodological limitations of bibliometric studies, they remain useful tools for evaluating the social and scientific importance of a selected discipline [13] considering the fact that they give an insight of the growth, size and distribution of scientific literature in the field of interest within a specified time frame [14]. To facilitate the understanding of on-going Lassa fever research output, social network analysis (SNA) of bibliometric data are usually used. Evaluation of research collaboration and its subsequent evolution over time is usually done using co-authorship based SNA as same is the most apparent and assessable indicator for collaboration [15, 16]. SNA metrics can highlight network patterns and identify its most influential participants [17]. Results of bibliometric analysis plays major role as proxy indicator of research cum development as well as in strategic planning [18].

This study was aimed at identifying Lassa fever research activities and to analyse the structure of the evolving Lassa fever research community network over time, and identify existing research collaborations and influential action. This study will be important to researchers, clinicians, research funders and health policy makers in order to adopt stringent policies regarding infectious disease in view of Lassa fever.

## Methods

### Data source

The SCOPUS database was used for this study. Scopus was selected as it is the largest abstract and citation database of peer-reviewed literature including: scientific journals, books and conference proceedings. Scopus index nearly 22,000 titles from over 5000 publishers, of which 20,000 are peer-reviewed journals in the scientific, medical, technical and social sciences (including the arts and humanities). Comparatively, Scopus is more comprehensive and user friendly to be used in biomedical discipline when compared to other bibliometric database for literature research, and it is well documented as the world’s largest database for abstract and citation information used in various bibliometric studies [19, 20]. We retrieved articles published from 1970 (year of the first record) to 2017 containing the descriptors “Lassa fever” limited to three fields: title, key word and/or abstract, using remote-downloading techniques. This study took into account all original articles, reviews, brief reports, letters to the editor, editorials, and more.

### Bibliometric indicators

The bibliometric indicators used in this study includes: Price’s productivity index, Price’s law, duplication time and annual growth rate, Lokta’s productivity level (PL), Price's transient index, Bradford zones, impact factor and co-authorship index.

Price’s Law [21] is broadly used indicator of productivity used in assessing the productivity of a particular discipline or country. It uses exponential growth evaluation which is an important feature of scientific productivity. To assess if a scientific production in Lassa fever research follows Price law of exponential growth, the generated data is modelled into linear adjustment according to the equation y = 0.6676x-1308 and exponential plot according to the equation y = 8E-32e^0.0373^. Price law is said to be fulfilled when the coefficient of determination of the exponential plot is greater than that of the linear plot.

Bradford’s law [22], was used to determine the distribution of scientific literature on Lassa fever in this study. Bradford’s law is a bibliometric indicator of dispersion of scientific literature. Bradford proposed concentric zones of productivity (Bradford zones) with decreasing density of information. He hypothesized each zone to contain similar number of documents. However, the number of journals that are produced increases from one zone to the next. Bradford’s postulated zones aids in identifying journals that are widely used in a specified discipline. The stratification of journals in the different Bradford zones are viz.: 1, n, n^2^… The number of articles is stratified into 3 groups of approximately same size in which one is the core zone while the other two are the peripheral zones.

We also used duplication time and annual growth as indicators of productivity of scientific literature. Duplication time and annual growth are associated with growth assessment. Duplication time refers to the time (years) it takes a subject to duplicate its production. On the other hand, annual growth refers to the value of the present growth in comparison with that of the previous year. The equation for the duplication time is viz.:

$$ D=\frac{Ln2}{b} $$

where b is the constant that relates the growth rate with the already acquired size of the discipline. The annual growth rate was calculated using the equation:

$$ \mathrm{R}=100\left({\mathrm{e}}^{\mathrm{b}}-1\right) $$

Lotka’s productivity index (PL) was used to assess productivity of authors. Lotka’s author distribution law was proposed on the basis of the number of published articles known as “quadratic inverse of scientific production” [23]. Lotka carried out quantitative evaluation of publication of authors and realized that there are large number of authors that publish few articles than the number that publish many. The law states that within the scientific community, the number of authors (A) that have published a specific number of articles (n) within a given period, that is A(n) authors equals the number of authors that have published a single article A(1) within the same time, divided by the square of n. It is represented mathematically as:

$$ A(n)=\frac{A(1)}{n^2} $$

According to Lotka’s index, authors are divided into 3 categories of productivity: those who published a single paper referred to as small producers, those who published between 2 to 9 papers who are referred to as mid-range producers and lastly those who published 10 or more papers, who are referred to as large producers.

Price’s transience index was used to assess the number of authors having a single publication. The calculation is given as a percentage of the quotient of authors with a single publication among all authors. It is expressed mathematically as:

$$ IT=\frac{authors\ with\ a\  single\ publication}{all\  author}\times 100 $$

Impact factor (IF) was used as an indicator of publication repercussion. Impact factor as a bibliometric indicator was developed by Institute for Scientific Information (Philadelphia, PA, USA). It is published yearly in the *Journal Citation Report* (JCR) section of Science Citation Index Expanded (SCI). The calculation takes into account the number of times the journal was cited in the source SCI within the two preceding years. The impact factor data of 2017 by JCR was used for this study.

Co-authorship index was used to determine level of collaboration in the publication of Lassa fever related documents.

The last indictor used in this study is the national participation index (PI) in overall scientific publication in Lassa fever and in the field of infectious diseases in world’s ten most productive countries in biomedical and health sciences during the period 1970–2017. Participation index reflects quotient between the number of documentation produced by a given country and the total number of documents obtained in the repertoire.

## Results

### Evaluation of global publication

Using the search criteria, we recovered 1101 research publications within the 47 years period (1970–2017). Of these, 67.67% (*n* = 745) were Original articles, while 17.35% (*n* = 191), 4.90% (*n* = 54), 2.27% (*n* = 25), 2.09% (*n* = 23), 1.82% (*n* = 20), 1.73% (n = 19), 1.18% (*n* = 13), 0.45% (n = 5), 0.36% (*n* = 4) and 0.18% (n = 2) were Reviews, Letters, Editorials, Notes, Short surveys, Book chapters, Conference papers, Articles in press, Errata and Books, respectively (Table 1).

**Table 1:** Contributing literature type

Document type	No of documents	%
Article	745	67.67
Review	191	17.35
Letter	54	4.90
Editorial	25	2.27
Note	23	2.09
Short Survey	20	1.82
Book Chapter	19	1.73
Conference Paper	13	1.18
Article in Press	5	0.45
Erratum	4	0.36
Book	2	0.18

**Fig. 1:**
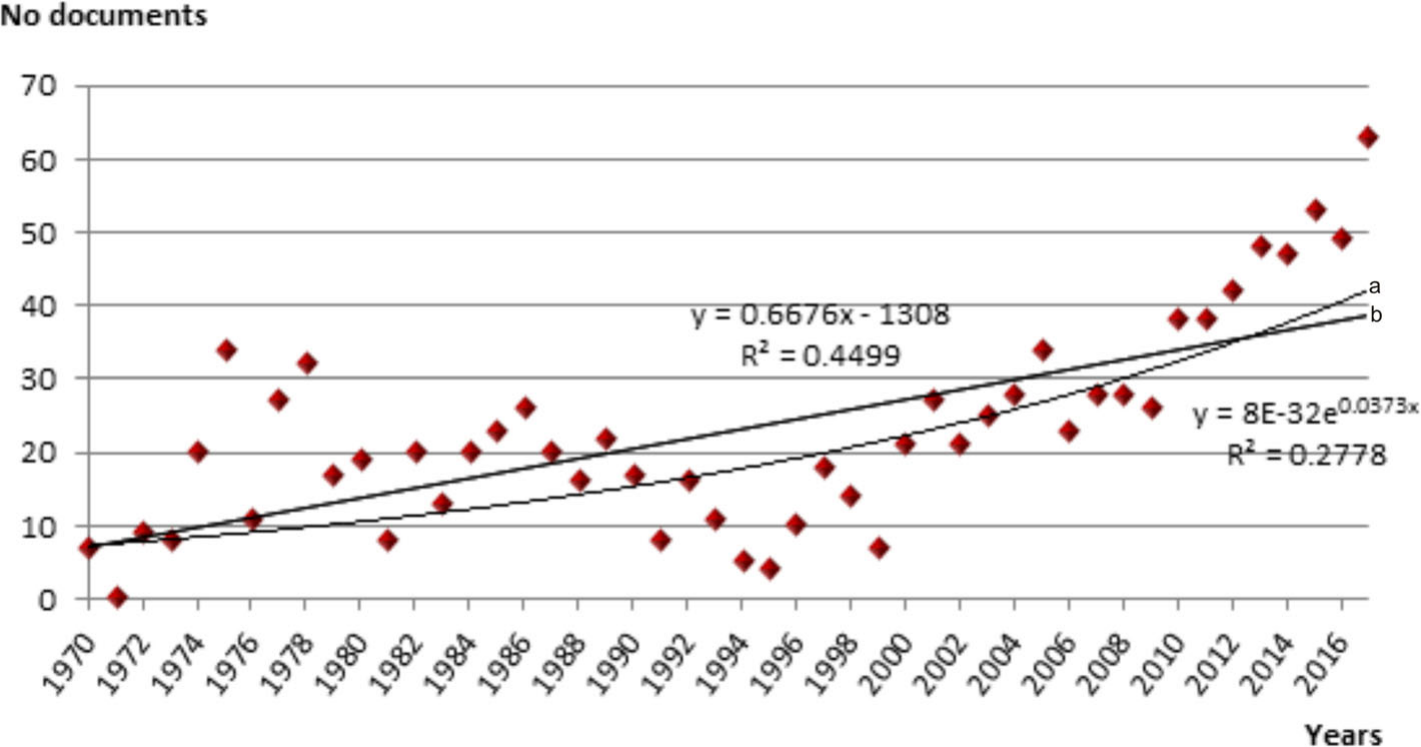
Chronological distribution of scientific literature on Lassa fever within the study period. **b** Linear trendline. **a** Exponential trendline

The chronological distribution of the publication showed that there has been notable increase in the number of articles generated in the area of Lassa fever research (Fig. 1). To determine whether the increase of scientific literature followed Price’s law, the obtained data were linearly adjusted in accordance with the equation y = 0.6676x-1308, and another adjustment in the exponential curve in accordance with the equation y = 8E-32e^0.037^. Hence, Price law is not fulfilled (*r* = 0.6707 in linear adjustment versus *r* = 0.5270 in exponential adjustment). This shows that growth of scientific literature in the area of Lassa fever research is in the linear growth stage.

**Fig. 2:**
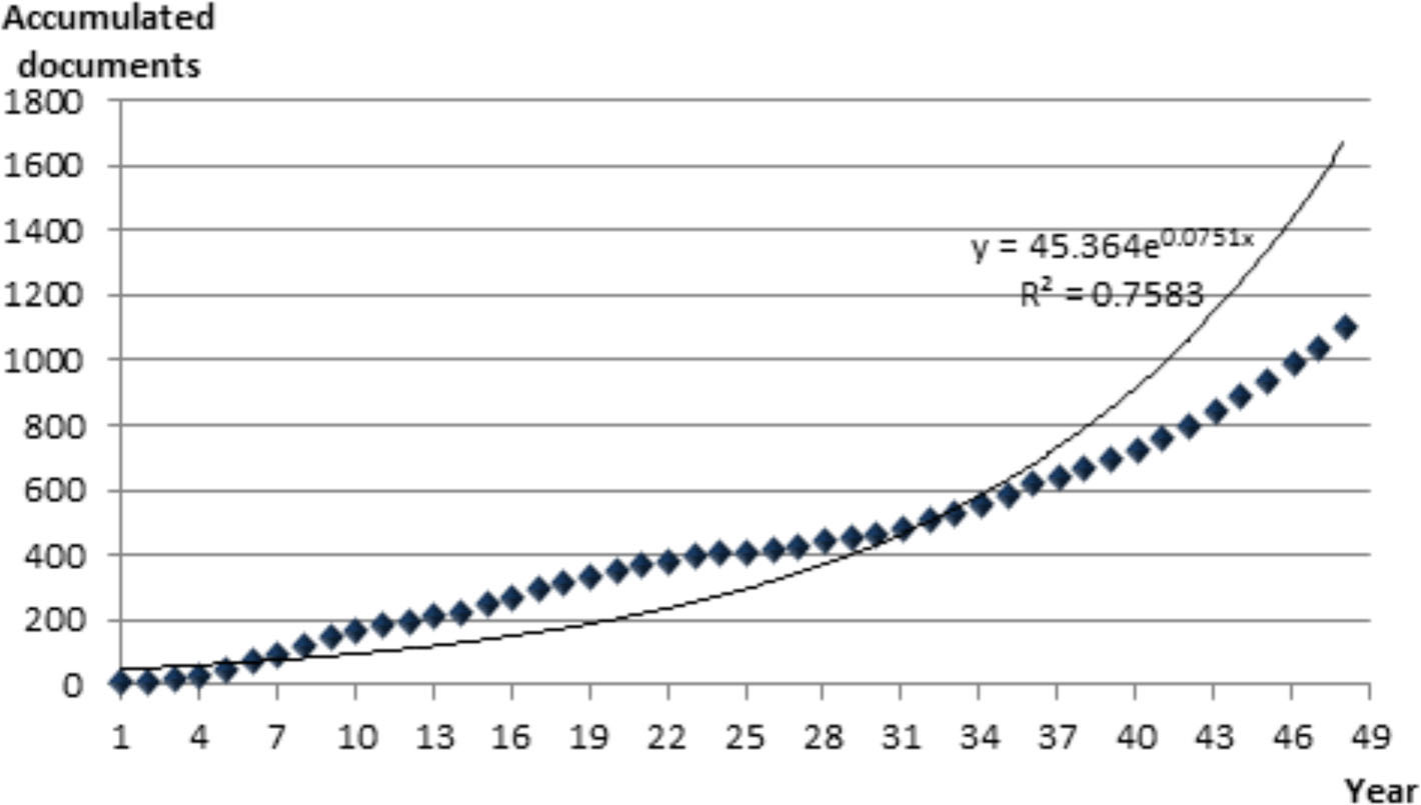
Temporal evolution of publication in Lassa fever. $D=\frac{Ln2}{b}=\frac{0.68904}{0.0751}=9.19$. Production doubles every 9.19 years

Figure 2 shows the temporal production of the literature publication. To calculate the duplication time, the dispersion graph was adjusted to the equation y = 45.365e^0.0751x^, and a determination coefficient of 0.87. The production covered 47 years. Hence, applying the equation for calculating duplication time, the result is 9.19 years. That means that production of scientific literature in area of Lassa fever doubles every 9.19 years.

Table 2 shows the stratification of the authors in groups according to their productivity level (PL). We observed that the largest group is made up of authors with a single publication (PL = 0), accounting for 78.89% whereas large producers (PL ≥ 1) with over 10 published papers accounted for 1.20%, being the least fraction of the group. Hence, price transient index that corresponds to occasional authors who have produced one paper is 78.89.

**Table 2:** Classification of authors based on productivity

	NP ≥ 1 (10 or more articles)	0 < NP < 1 (2–9 articles)	NP = 0 (1 article)	Total
Number of authors	38	633	2508	3179
% Authors	1.20	19.91	78.89	100.00

Table 3 shows the distribution of journals per Bradford zone. Nineteen (4.34%) of the journals made up the core zone, while 74 (16.89%) and 345 (78.77%) made up the zone 1 and 2.

**Table 3:** Bradford division of journals

	No of journals	% of journals	No of articles	% of articles	Bradford multiplier
Core	19	4.34	372	33.79	
Zone 1	74	16.89	332	30.15	3.89
Zone 2	345	78.77	397	36.06	4.66
Total	438	100.00	1101	100.00	4.27

**Fig. 3:**
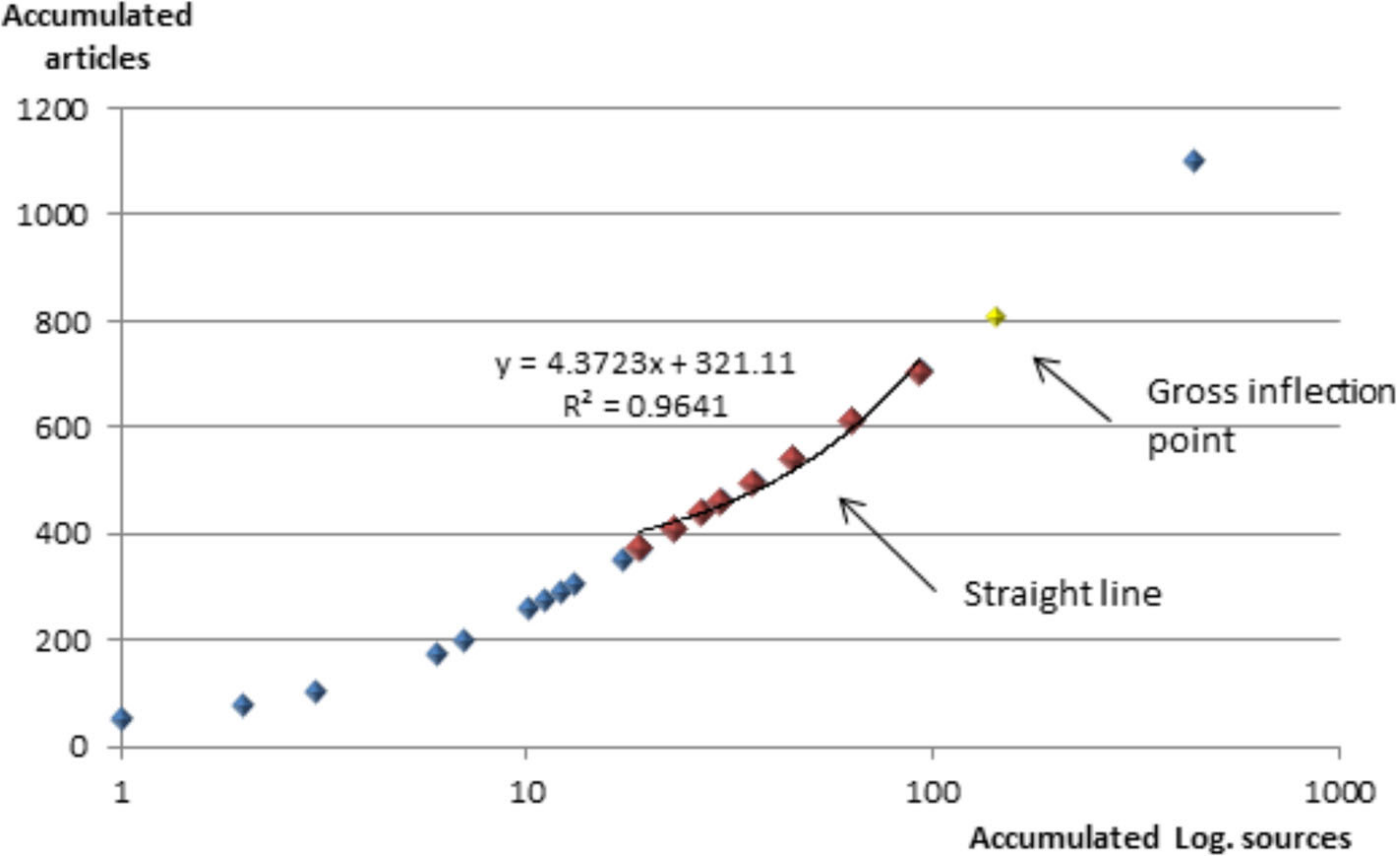
Bradford distribution, global data

Figure 3 shows Bradford distribution, global data. This is a semi – logarithm diagram of the aggregate number of articles *versus* the aggregate number of journals (r). The straight zone has been considered for *r* = 19 to *r* = 93, adjusted to y = 4.3723x + 321.11 equation with a high value of the determination coefficient (0.9919).

### Characteristics of collaboration

The 1101 published articles recorded in this study were produced by 3179 authors with a mean co-authorship of 4.59. The document with the most authors had 84 signatures and the most frequent number of signature was 1 (Table 4).

**Table 4:** Analysis of collaboration among authors

Signing authors	No. Of documents	Aggregate	Documents (%)
84	1	84	0.091
72	1	72	0.091
51	1	51	0.091
50	1	50	0.091
35	2	70	0.182
33	1	33	0.091
31	1	31	0.091
30	1	30	0.091
27	1	27	0.091
26	1	26	0.091
25	2	50	0.182
22	2	44	0.182
20	2	40	0.182
19	2	38	0.182
18	3	54	0.272
17	2	34	0.182
16	6	96	0.545
15	8	120	0.727
14	8	112	0.727
13	13	169	1.181
12	18	216	1.635
11	17	187	1.544
10	31	310	2.816
9	35	315	3.179
8	33	264	2.997
7	42	294	3.815
6	71	426	6.449
5	74	370	6.721
4	104	416	9.446
3	134	402	12.171
2	148	296	13.442
1	335	335	30.427

### Analysis of sources with highest publication

Table 5 shows the top 10 journals used for the dissemination of research publications on Lassa fever with their corresponding impact factors (IFs) according to JCR of 2016 and the participation index (PI) of the journals on total database in the analyzed period. All the journals have impact factor with 6 and 3 of them having impact factors greater than 4 and 2, respectively.

**Table 5:** Analysis of the top 10 sources with the largest number of publications^a^

Source	No of documents	Productivity Index	Impact Factor	Country of origin	Abbreviated Journal Title
*Journal of Virology*	51	4.63	4.663	United States	J. Virol.
*Transactions of the Royal Society of Tropical Medicine and Hygiene*	27	2.45	2.279	England	Roy. Soc. Trop. Med. Hyg.
*Voprosy Virusologii*	25	2.27	0.160^b^	Russia	Vopr. Virusol.
*American Journal of Tropical Medicine and Hygiene*	24	2.18	2.549	United States	Am. J. Trop. Med. Hyg.
*Bulletin of the World Health Organization*	24	2.18	4.939	Switzerland	Bull. World Health Organ.
*Emerging Infectious Diseases*	24	2.18	8.222	United States	Emerg. Infect. Dis
*The Lancet*	22	2.00	47.831	England	Lancet
*British Medical Journal*	20	1.82	20.785	England	BMJ – Br. Med. J.
*Journal of Infectious Diseases*	20	1.82	6.273	United States	J. Infect. Dis.
*PLoS Neglected Tropical Diseases*	20	1.82	3.834	United States	Plos Neglect. Trop. Dis.

^a^Data from the Journal Citation Report

^b^Data 1998

### Productivity of countries

Countries that are most productive in publishing documents on Lassa fever were listed in Table 6. The United States was the most productive (*n* = 450) whose PI is 40.87, followed by United Kingdom (*n* = 117; PI = 10.63), Germany (*n* = 96; PI = 8.72), Nigeria (*n* = 65; PI = 4.90) and Sierra Leone (*n* = 54; PI = 4.90).

**Table 6:** Top 20 most productive countries in Lassa fever research

Country	No of documents	%
United States	450	40.87
Undefined	261	23.71
United Kingdom	117	10.63
Germany	96	8.72
Nigeria	65	5.90
Sierra Leone	54	4.90
France	44	4.00
Canada	32	2.91
Switzerland	32	2.91
Netherlands	22	2.00
Guinea	21	1.91
Japan	21	1.91
Sweden	18	1.63
South Africa	17	1.54
Belgium	16	1.45
Italy	12	1.09
Liberia	12	1.09
Australia	10	0.91
India	10	0.91
China	9	0.82

However, if we consider the productivity of the most productive countries on Lassa fever research in relation to their overall production in the field of infectious disease, only United States, United Kingdom, Germany, France and Canada of the top 10 most productive countries in the field of infectious disease were active (among the top 10 in biomedical research) in the production of scientific literature on Lassa fever. United States, and United Kingdom were leading consistently in all areas of infectious diseases (Lassa fever, Tuberculosis, AIDS) and Medicine (Table 7).

**Table 7:** Relationship between production of scientific literature on Lassa fever and total production in some fields of infectious diseases in world 10 most productive countries in biomedical and health sciences

Country	IP^a^ Medicine^b^	IP Health Professions^a^	IP Pharmacology, Toxicology and Pharmaceutics^a^	IP Lassa Fever^c^	IP Tuberculosis^b^	IP AIDS^b^
United States	25.51	49.80	15.01	40.87	18.38	33.76
United Kingdom	8.07	14.43	4.14	10.63	8.04	7.43
Germany	6.10	1.36	3.17	8.72	3.78	3.81
China	5.86	0.39	1.,37	0.82	3.25	3.17
Japan	4.29	0.65	3.12	1.91	4.29	2.12
France	3.84	0.66	1.71	4.00	4.50	3.72
Canada	3.64	6.27	1.44	2.91	2.22	3.32
Italy	3.08	0.74	1.95	1.09	2.84	2.93
Australia	2.79	5.91	1.18	0.91	1.53	2.43
Spain	2.78	0.65	1.34	0.64	2.87	2.10

^a^Participation Index

^b^Scimago Journal & Country Rank

^c^Data 1970–2017

### Productivity of institutions

Table 8 shows the top 20 most productive institutions in Lassa fever research. Centre for Disease Control (USA) was the most productive institution (10.26%; *n* = 13) followed by Bernhard Nocht Institute fur Tropenmedizin Harmburg, Germany (Bernhard Nocht Institute for Tropical Medicine) (5.54%; *n* = 16), U.S. Army Medical Research Institute of Infectious Disease (4.81%; *n* = 53), Scripps Research Institute, USA (4.72%; *n* = 52) and others. Sixty five percent (65%) of the top 20 most productive institutions were in the United States, whereas the rest were from Germany, United Kingdom, Sierra Leone, Nigeria and France. Seven of the institutions (35%) are government agencies (35%), while 4, 3, 3, 2 and 1% are public/ Government Universities, nonprofit/ governmental organizations, private universities, private laboratories and government hospital, respectively.

**Table 8:** Top 20 most productive institutions in Lassa fever research

Instiution	Country of residence	No of documents	%
Centers for Disease Control and Prevention	USA	113	10.26
Bernhard Nocht Institut fur Tropenmedizin Hamburg	Germany	61	5.54
U.S. Army Medical Research Institute of Infectious Diseases	USA	53	4.81
Scripps Research Institute	USA	52	4.72
UT Medical Branch at Galveston	USA	44	4.00
National Center for Infectious Diseases	USA	34	3.09
National Institutes of Health. Bethesda	USA	31	2.82
Tulane University	USA	28	2.54
UCL (University College London)	UK	27	2.45
Kenema Government Hospital	Sierra Leone	24	2.18
Institute of Human Virology	Nigeria	22	2.00
University of Ibadan	Nigeria	18	1.63
Universitat Marburg	Germany	18	1.63
Irrua Specialist Teaching Hospital	Nigeria	16	1.45
Tulane University School of Public Health and Tropical Medicine	USA	15	1.36
National Center for Emerging and Zoonotic Infectious Diseases	USA	15	1.36
Zalgen Labs	USA	14	1.27
Emory University	USA	14	1.27
Institut Pasteur Paris	France	14	1.27
Viral and Rickettsial Zoonoses Br.	USA	14	1.27

### Productivity of authors

Table 9 shows top 6 most productive authors in Lassa fever research. Five authors in the top 6 most productive list are from United States and were affiliated with University of Texas Health Science Centre, Zalgens Labs, University of Maryland and Centre for Predictive Medicine for Biodefence and Emerging Infectious Disease. The other is from Germany and affiliated to Bernhard Nocht Institute for Tropical Medicine.

**Table 9:** Top 6 authors with the most publications related to Lassa fever

Author	No of Documents	% Documents	h-index	Country	Afiliattion	Cited	% Cited	Article/cited
McCormick J.B.	39	3.54	56	United States	University of Texas Health Science Center at Houston	2174	9.40	55.74
Günther S.	35	3.18	53	Germany	Bernhard Nocht Institut fur Tropenmedizin Hamburg	1329	5.75	37.97
Garry R.F.	31	2.82	37	United States	Zalgen Labs. Germantown	523	2.26	16.87
Salvato M.S.	25	2.27	32	United States	University of Maryland	748	3.23	29.92
Lukashevich I.S.	22	2.00	24	United States	Center for Predictive Medicine for Biodefense and Emerging Infectious Diseases	806	3.49	36.64
Grant D.S.	20	1.82	12	United States	Zalgen Labs	265	1.15	13.25

### Citation analysis of articles

Table 10 shows top 10 most cited articles. Manually analyzing the articles showed that the top cited article mentioned Lassa fever in passing while the other publications dealt on public health concern, description, diagnosis and management of Lassa fever.

**Table 10:** Top 10 most cited articles

Article	Authors	Source	Cited	%
Legionnaires’ Disease: Description of an Epidemic of Pneumonia	Fraser, D.W., Tsai, T.R., Orenstein, W., (...), Shepard, C.C., Brachman, P.S.	*New England Journal of Medicine*	1071	4.63
Lassa Fever	McCormick, J.B., King, I.J., Webb, P.A., (...), Elliott, L.H., Belmont-Williams, R.	*New England Journal of Medicine*	549	2.37
Identification of α-dystroglycan as a receptor for lymphocytic choriomeningitis virus and Lassa fever virus	Cao, W., Henry, M.D., Borrow, P., (...), Campbell, K.P., Oldstone, M.B.A.	*Science*	429	1.86
Social and environmental risk factors in the emergence of infectious diseases	Weiss, R.A., McMichael, A.J.	*Nature Medicine*	305	1.32
Rodent-borne diseases and their risks for public health	Meerburg, B.G., Singleton, G.R., Kijlstra, A.	*Critical Reviews in Microbiology*	256	1.11
Importance of Aeromonas Sobria in Aeromonas Bacteremia	Janda, J.M., Brenden, R.	*Journal of Infectious Diseases*	252	1.09
The small RING finger protein Z drives arenavirus budding: Implications for antiviral strategies	Perez, M., Craven, R.C., De la Torre, J.C.	*Proceedings of the National Academy of Sciences of the United States of America*	218	0.94
Molecular mechanisms of action of ribavirin	Patterson, J.L., Fernandez-Larsson, R.	*Reviews of Infectious Diseases*	217	0.94
Prospects for Treatment of Viral Hemorrhagic Fevers with Ribavirin, a Broad-Spectrum Antiviral Drug	Huggins, J.W.	*Reviews of Infectious Diseases*	209	0.90
Lassa fever	McCormick, J.B., Fisher-Hoch, S.P.	*Current Topics in Microbiology and Immunology*	192	0.83

## Discussion

The document type utilized mostly by authors in studied repertoire is mostly original article accounting approximately 67.67% of all publications. This shows that the subject matter is clinical research or experimental.

This study observed a linear growth of scientific literature in Lassa fever with average annual increase of 15.73% showing non fulfillment of Price’ Law of growth. This pattern of growth of scientific literature is at variance with previous reports in other areas of medical research [13, 24, 25]. This finding shows poor growth and low interest. This poor research output could be due to the limitation imposed by the Biosafety level 4 requirement for Lassa fever research [26]. More so, the escalating funding for infectious diseases such as HIV/AIDS may have created a shortage of funding for other diseases of regional burden such as Lassa fever [27]. Funding has been reported to have a positive influence on research output and citations for a particular disease [28].

This study also showed high transience rate of 78.89% indicating that the authors were mainly occasional publishers. This could be interpreted as low productivity or an indication of presence of researchers of other related specialties that have sporadically published in this field [29]. Only 1.20% of the authors were large producers. This shows that a large part of the scientific literatures emanated from a small number of researchers.

The proportion of papers signed by more than one author was 69.57%. This showed high level of co-operation among researchers. The mean co-authorship index is 2.88. Collaboration between authors indicates teamwork. Team work becomes essential considering multifaceted nature of contemporary research as well as cost implications.

Bradford analysis of the studied repertoire showed 19 journals in the core zone. This means that only 19 journals produced 33.79% of the published literatures. This shows a high concentration of publications by a small amount of journals. Of this number, *Journal of Virology* amassed the highest number of publication (51) accounting for 4.63% of all publications. The top 10 journals were mainly those dedicated to virology, infectious diseases or tropical diseases. Only *British Medical Journal* and *The Lancet* were the multidisciplinary journals.

United States of America and United Kingdom topped the ranking of research publication in Lassa fever. Both dominated research output in Lassa fever and accounted 64.57% of the total research output. This observation is consistent with previous research in other biomedical research [13, 24, 30]. The United States alone accounted for 40.87% of all research output in this area of study. It could not be far from the fact that these two countries are home to the pharmaceutical companies that manufacture ribavirin, an agent approved for the management of Lassa fever: Copegus, by Gentech Laboratories USA, UK- member of Roche group; Rebetol, by Merck Sharp and Dome, USA, UK; Ribasphere, by Kadmon Corporation, USA. The United States also houses the Zalgen Lab that deals on diagnostic logistics in Lassa fever. Same institution is responsible for production of the rapid test kit ReLASV for Lassa fever. Nigeria and Sierra Leone were the only African countries present on the list. These two countries have been reported to be endemic with Lassa fever [4, 9]. There would be a relationship with this endemic disease and the public health concern translated into scientific production [31]. More so, the fact that Nigeria was the first country where the disease was identified [2] may be a contributing factor to Nigeria’s commitment. In relation to top 10 most productive in biomedical sciences, the interest of China in AIDS and tuberculosis and lack of same on Lassa fever was remarkable. Similar trend was also found in Australia and Spain. Institutions in the United Sates were the leading organizations in Lassa fever research which goes further to corroborate the finding that USA tops in global publication in Lassa fever. More than half (60%) of the institution were located in United States. The remaining slot went to other European countries such as Germany, and UK. This suggests that creating first-class research institutes is fundamental to improving academic level of a country. Sierra Leone and Nigeria were the only African country that housed institution that ranked among the first top 20 institutions (10/20). Kenema Government Hospital Sierra Leone is a center of international effort to combat Lassa fever with support from World Health Organization and UNAMSIL (United Nations Mission in Sierra Leone). Also, Sierra Leone is among the three members (Guinea, Liberia, Sierra Leone) of the Manu River Union Lassa fever Network established in partnership with World Health Organization, United States Foreign Disaster Assistance and United Nations [32]. Institute of Human Virology Nigeria is a leading local non-governmental organization addressing HIV/ AIDS crisis in Nigeria that has expanded its services to other infectious diseases [33]. University of Ibadan is a government owned Nigerian University whose mission is to expand the frontiers of knowledge through provision of excellent conditions for learning and research.

McCormick JB, Gunthers S, Garry RF, Salvato MS, Lukashevich IS and Grant DS were the top 6 authors who published the most studies in the Lassa fever research. McCormick JB focused on the treatment, epidemiology, general characterization of the disease and diagnosis while Gunters S, focused on the molecular characterization of the disease, and diagnosis. Garry RF focused on treatment and diagnosis of the disease, while Salvato MS dedicated her effort to molecular characterization of the disease, diagnosis and vaccine prototype production. Grant DS made impact in the area of diagnosis, origin and evolution of the disease, and epidemiology of the disease.

The total number of the cited articles within the studied repertoire was 23,125 giving an average citation count of 21 per article. The article “Legionnaire Disease: description of an epidemic of pneumonia” published by *New England Journal of Medicine* was the most cited article. On critical analysis, it is quite ironic that an article devoted to epidemiology of Legionaires disease made the highest number of citation on Lassa fever study analysis. These citations emanated from a two-line sentence from the author summary of the article referring to discovery of Lassa fever and Ebola made possible via epidemiology investigations. This scenario is one of those limitations of citations as a bibliometric impact parameter. The wide circulation of the journal may have leveraged it to achieve same at the cost of content relating to the topic in question. The second most cited article was an article on clinical trial on the effectiveness of ribavirin in preventing mortality in Lassa fever also published by *New England of Medicine*. This goes further to buttress how active the journal is as well as justify the high impact factor of the journal (55.5). The third and fourth articles were on cellular receptors of Lassa fever virus and social and environmental risk factors in the emergence of infectious diseases. It is worthy of note that McCormick is the author of the second, and tenth articles as well as the most productive author. Only one of the journals (*New England Journal of Medicine*) is an open access journal (with 6 months embargo). The others (*Science*, *Nature Medicine*, *Critical Reviews in Microbiology*, *Journal of Infectious Diseases*, *Proceedings of the National Academy of Sciences of the United States of America* and *Current Topics in Microbiology and Immunology*) are toll access journals while *Reviews in Infections Disease* is hybrid. These journals were all well-established journals. This shows that open access and toll access are uniformly favored in citation for well-established journals, but the impact tend to peak when such established journal is open access as in the case of *New England Journal of Medicine*.

### Limitations

However, this study contains some limitations which are inherent in bibliometric analysis. This study includes papers from SCOPUS database. The criteria set by the databases themselves determine the subsequent development of the studied materials [34]. We might have excluded papers on Lassa fever if the authors have not put our study inclusion descriptors in the titles or as key words. More so, local journals that are not indexed in SCOPUS during the study period were also not included in our study.

## Conclusion

Despite the above named limitations, this study has been able to illuminate on the characteristics of Lassa fever research output from 1970 to 2017. There is a slow growth in research activities related to Lassa fever from 1970 to 2017. This research demonstrated that the growth of Lassa fever related literature favors a linear path rather than exponential unlike other biomedical fields. The bulk of publications in the field of Lassa fever research are published by high income countries such as the United States of America and United Kingdom. This study showed that majority of the most productive institutions were resident in the USA. Bulk of the articles were produced by very few of the participating authors. Thus, this study provides a helpful reference for medical virologists, epidemiologist, policy decision makers, academics and Lassa fever researchers.

## Abbreviations

IF: Impact factor
JCR: Journal citation report
LASV: Lassa virus
PI: Participation index
PL: Productivity level
SCI: Science citation index
SNA: Social network analysis
SNHL: Sensorineural hearing loss
